# Posterior cruciate ligament management in medial pivot total knee arthroplasty: a systematic review and meta-analysis

**DOI:** 10.1186/s13018-026-06677-8

**Published:** 2026-03-24

**Authors:** Filippo Migliorini, Marco Pilone, Luise Schäfer, Raju Vaishya, Giorgio Moretti, Thomas Mendel, Gennaro Pipino, Nicola Maffulli

**Affiliations:** 1 Department of Trauma and Reconstructive Surgery, University Hospital of Halle, Martin-Luther University Halle-Wittenberg, Ernst-Grube-Street 40, 06097 Halle (Saale), Germany; 2 Department of Orthopaedic and Trauma Surgery, Academic Hospital of Bolzano (SABES-ASDAA), via Lorenz Böhler 5, 39100 Bolzano, Italy; 3 Department of Life Sciences, Health, and Health Professions, Link Campus University, Via del Casale di San Pio V, 00165 Rome, Italy; 4 Department of Orthopaedic and Trauma Surgery, Eifelklinik St.Brigida, Kammerbruchstr. 8, 52152 Simmerath, Germany; 5 Residency Program in Orthopaedics, University of Milan, Milan, Italy; 6 Department of Orthopaedics and Joint Replacement Surgery, Indraprastha Apollo Hospital, Sarita Vihar, New Delhi, 110076 India; 7 Department of Trauma and Reconstructive Surgery, BG Klinikum Bergmannstrost Halle GmbH, Halle (Saale), Germany; 8 Department of Orthopaedics, Villa Erbosa Hospital, Bologna, Italy; 9 Department of Trauma and Orthopaedic Surgery, Faculty of Medicine and Psychology, University “La Sapienza” of Rome, Via di Grottarossa 1035, 00189 Rome, Italy; 10 School of Pharmacy and Bioengineering, Keele University Faculty of Medicine, Stoke on Trent, ST4 7QB UK; 11 Centre for Sports and Exercise Medicine, Queen Mary University of London, Barts and the London School of Medicine and Dentistry, Mile End Hospital, 275 Bancroft Road, London, E1 4DG UK

**Keywords:** Medial congruency, Biomechanics, Replacement, Joint, Revision, Prosthesis, Kinematics

## Abstract

**Introduction:**

Medial pivot total knee arthroplasty (TKA) was designed to replicate physiological tibiofemoral kinematics, yet the role of posterior cruciate ligament (PCL) management in this setting remains controversial. This systematic review and meta-analysis aimed to compare the clinical and functional outcomes, as well as revision rates, between PCL retention and resection in medial pivot TKA.

**Methods:**

A comprehensive search of PubMed, Web of Science, Embase, and Google Scholar was conducted in August 2025, following the PRISMA guidelines. Comparative and non-comparative clinical studies reporting outcomes of medial pivot TKA with either PCL retention or resection were included. Outcomes of interest were Knee Society Score (KSS) and its functional subscale (KSS-F), Oxford Knee Score (OKS), Western Ontario and McMaster Universities Osteoarthritis Index (WOMAC), Forgotten Joint Score (FJS), range of motion (ROM), and revision rates.

**Results:**

Twenty-seven studies involving 3380 patients were included, of whom 1209 underwent medial pivot total knee arthroplasty (TKA) with posterior cruciate ligament (PCL) retention and 2171 with PCL resection. Baseline characteristics were comparable, except for follow-up duration and sex distribution. At the final follow-up, both groups achieved similar outcomes for the Knee Society Score (KSS), Oxford Knee Score (OKS), Western Ontario and McMaster Universities Osteoarthritis Index (WOMAC), Forgotten Joint Score (FJS), and range of motion (ROM). The PCL-retained cohort exhibited slightly higher functional and clinical scores, yet the magnitude of difference remained below the threshold of minimal clinical significance. Revision rates were low and comparable between the two groups.

**Conclusion:**

PCL retention and resection in medial pivot TKA yield statistically different but clinically equivalent results. The small numerical advantages observed for the retained group in certain functional outcomes do not appear to represent a meaningful clinical improvement. Both strategies can therefore be considered viable, and adequate alignment, balancing, and soft-tissue management remains pivotal. Further high-quality comparative studies involving well-matched populations are warranted to clarify whether subtle functional trends associated with PCL retention have consistent long-term clinical significance.

**Level of evidence:**

Level III.

## Introduction

Total knee arthroplasty (TKA) is a common procedure [1, 2]. The outcomes of TKA are typically good, with improvements in function and patient quality of life [3, 4]. TKA has advanced significantly in recent years, with various designs aimed at enhancing patient satisfaction [5, 6]. Medial-pivot (MP) TKA is designed to reproduce native tibiofemoral motion by creating a highly congruent “ball-in-socket” medial compartment that acts as the centre of axial rotation while permitting controlled lateral rollback, thereby aiming to restore physiological coupling between flexion and internal tibial rotation [7, 8]. The use of more conforming articular surface geometries reduces polyethilene wear and improves patellar tracking. [9–11]. Compared to traditional designs, MP prostheses may offer enhanced stability and more physiological load distribution, especially in high-demand and younger patients [12]. The posterior cruciate ligament (PCL) plays a crucial role in knee stability and kinematics, contributing to posterior femoral rollback and rotational control during flexion [13–16]. Emerging evidence specific to MP indicates that retaining the PCL can increase passive internal tibial rotation without inducing anterior lift-off at 90° when MP is implanted with calliper-verified kinematic alignment, suggesting a stabilising and rotational-guiding role for the ligament in a medial-pivot construct [17, 18]. Despite consistent kinematic rationale, its influence on surgical outcomes remains controversial, and the decision to preserve or sacrifice the PCL remains largely based on surgeon preference [13, 19–22]. This ongoing debate has led to heterogeneous surgical practices and inconsistent clinical outcomes [23, 24]. As surgical techniques evolve toward more personalised approaches and kinematic alignment, a better understanding of the role of the PCL in medial pivot TKA becomes increasingly important [25, 26]. Furthermore, patient-reported outcome measures (PROMs) have become essential in assessing the success of TKA, as they capture subjective perceptions of pain, stability, and function that may not be fully reflected by clinical scores alone [27–29]. This systematic review and meta-analysis compares clinical and functional outcomes and revision rates between PCL-retaining and PCL-sacrificing MP TKA. By synthesising existing evidence, this study seeks to clarify whether preserving the PCL offers significant advantages over its resection in the context of MP designs, thereby informing surgical decision-making. Standardising the approach to PCL management could thus lead to more predictable outcomes and improved patient satisfaction [30].

## Methods

### Eligibility criteria

All clinical studies evaluating medial pivot TKA performed with either PCL retention or resection that reported at least one relevant clinical or functional outcome were eligible for inclusion. Publications in English, German, French, Italian, or Spanish were reviewed. According to the Oxford Centre for Evidence-Based Medicine [31], studies corresponding to evidence levels I to III were included in the meta-analysis, while non-comparative level IV studies contributed to the qualitative synthesis only. Only studies with a minimum of 12 months of follow-up were included to ensure adequate evaluation of clinical outcomes and early implant survivorship. Case studies, narrative reviews, editorials, expert opinions, and letters were excluded. Preclinical research, including cadaveric dissections, animal experiments, computational models, and purely biomechanical testing, was not considered. Studies without quantifiable clinical outcomes relevant to the research question were excluded from the final analysis.

### Search strategy

This systematic review was conducted in line with the Preferred Reporting Items for Systematic Reviews and Meta-Analyses (PRISMA) 2020 guidelines [32]. To maintain a structured methodology and ensure transparency, the following PICO framework was applied:

- Problem: end-stage knee osteoarthritis;
- Intervention: medial pivot TKA;
- Comparison: PCL retention vs. resection;
- Outcomes: functional scores, range of motion, complications, and revision rates.

A comprehensive literature search was performed in August 2025 using PubMed, Web of Science, Google Scholar, and Embase. No time restrictions were applied. The complete search strategies, including search strings and Medical Subject Headings (MeSH) for each database, are presented in Table 1.

**Table 1:** MeSH

Database	Search strategy (MeSH terms and keywords)
PubMed	("Arthroplasty, Replacement, Knee"MeSH Terms OR "total knee arthroplasty" OR "total knee replacement" OR TKA) AND (("Medial Pivot"All Fields) OR "medial-pivot" OR "pivot knee" OR "medial congruent") AND (“posterior cruciate ligament”MeSH Terms OR PCL OR “cruciate retaining” OR “cruciate resecting” OR “CR” OR “PS”)
Web of Science	TS = ("total knee arthroplasty" OR "total knee replacement" OR TKA) AND TS = ("medial pivot" OR "medial-pivot" OR "pivot knee") AND TS = (“posterior cruciate ligament” OR PCL OR “cruciate retaining” OR “cruciate sacrificing” OR CR OR PS)
Google Scholar	"total knee arthroplasty" AND ("medial pivot" OR "medial-pivot" OR "pivot knee") AND (“posterior cruciate ligament” OR PCL OR “cruciate retaining” OR “cruciate sacrificing”)
Embase	('total knee arthroplasty'/exp OR 'total knee replacement' OR TKA) AND ('medial pivot' OR 'medial-pivot' OR 'pivot knee') AND (‘posterior cruciate ligament’/exp OR PCL OR ‘cruciate retaining’ OR ‘cruciate sacrificing’ OR CR OR PS)

### Selection and data collection

Two reviewers (F.M. and L.S.) independently screened all records identified through the database search. Titles and abstracts were assessed using the predefined eligibility criteria. If relevance could not be confirmed based solely on the abstract, the full text was retrieved and evaluated. To ensure completeness, reference lists of all included studies were manually searched for additional eligible publications. Disagreements during study selection were resolved by discussion; if necessary, a senior author (N.M.) acted as arbiter to reach a final decision.

### Data items

Two reviewers (F.M. and L.S.) independently extracted the data from all included studies. Baseline information included first author, year of publication, journal, study design, follow-up duration, number of patients, and mean age. Where available, the mean body mass index (BMI) was also recorded. Details of the implant type (resection and retention of PCL) were collected. Data on the following PROMs were extracted: Knee Society Score (KSS) and its functional subscale (KSS-F), the Western Ontario and McMaster Universities Osteoarthritis Index (WOMAC), the Forgotten Joint Score (FJS) and the Oxford Knee Score (OKS). Data on the range of motion (ROM) were also collected. Any complications or revisions were recorded. The threshold of the minimally clinically important difference (MCID) was: 5 points for the KSS, 10 for the KSS-F, 10 for the WOMAC, 14 for the FJS, and 5 for the OKS [29]. All data were compiled in Microsoft Excel (version 16.0, Microsoft Corporation, Redmond, WA, USA).

### Assessment of the risk of bias and quality of the recommendations

The risk of bias was assessed following the Cochrane Handbook for Systematic Reviews of Interventions guidelines [33]. Two reviewers (F.M. and L.S.) independently evaluated the risk of bias in the included studies. Disagreements were solved in consultation with a third senior author (N.M.). RCTs were appraised using the revised Risk of Bias assessment tool (RoB2) [34, 35] of the Cochrane tool for assessing Risk of Bias in randomised trials (RoB) [36], considering potential bias from the randomisation process, deviations from intended interventions, missing outcome data, outcome measurement, and bias in the selection of the reported result. The risk of bias in non-randomised studies was evaluated using the Risk of Bias in Non-randomised Studies of Interventions (ROBINS-I) tool [37] across seven domains. These covered potential confounding factors, patient selection, classification of interventions, deviations from intended interventions, missing data, outcome measurement, and selective reporting. The ROBINS-I visualisation was produced with the Robvis software (Risk-of-Bias VISualization, Bristol, UK) [38].

### Synthesis methods

The statistical analyses were performed by the main author (F.M.) following the recommendations of the Cochrane Handbook for Systematic Reviews of Interventions [39]. For descriptive statistics, the IBM SPSS software version 25 (International Business Machines Corporation, Armonk, USA) was used. For the analysis of the baseline comparability, the mean difference (MD) effect measure and the t-test were used. Values of *P* < 0.05 indicate statistically significant differences between groups in the baseline demographics. For the comparisons of the outcomes of interest at the last follow-up, the MD and the odds ratio (OR) effect measures were used for continuous and dichotomic data. Standard error was also calculated. The confidence interval was set at 95% in all comparisons. Values of *P* < 0.05 were considered statistically significant. For the meta-analyses, the Review Manager software version 5.3 (The Nordic Cochrane Collaboration, Copenhagen) was used. The paired t-test was performed with values of *P* < 0.05 considered statistically significant. For continuous data, the inverse variance method with the mean difference (MD) effect measure was used. For binary data, the Mantel–Haenszel method with odds ratio (OR) effect measure was used. The confidence interval (CI) was set at 95% in all the comparisons. Heterogeneity was evaluated through Higgins-I^2^ and χ^2^ tests. If P_χ2_ > 0.05, no statistically significant heterogeneity was found. If P_χ2_ < 0.05, the heterogeneity was evaluated following the values of the Higgins-I^2^. If the Higgins-I^2^ test > 50% high heterogeneity was found. A fixed effect model was set as the default. If high heterogeneity was detected, a random model effect was used. Overall values of *P* < 0.05 were considered statistically significant.

## Results

### Study selection

**Fig. 1:**
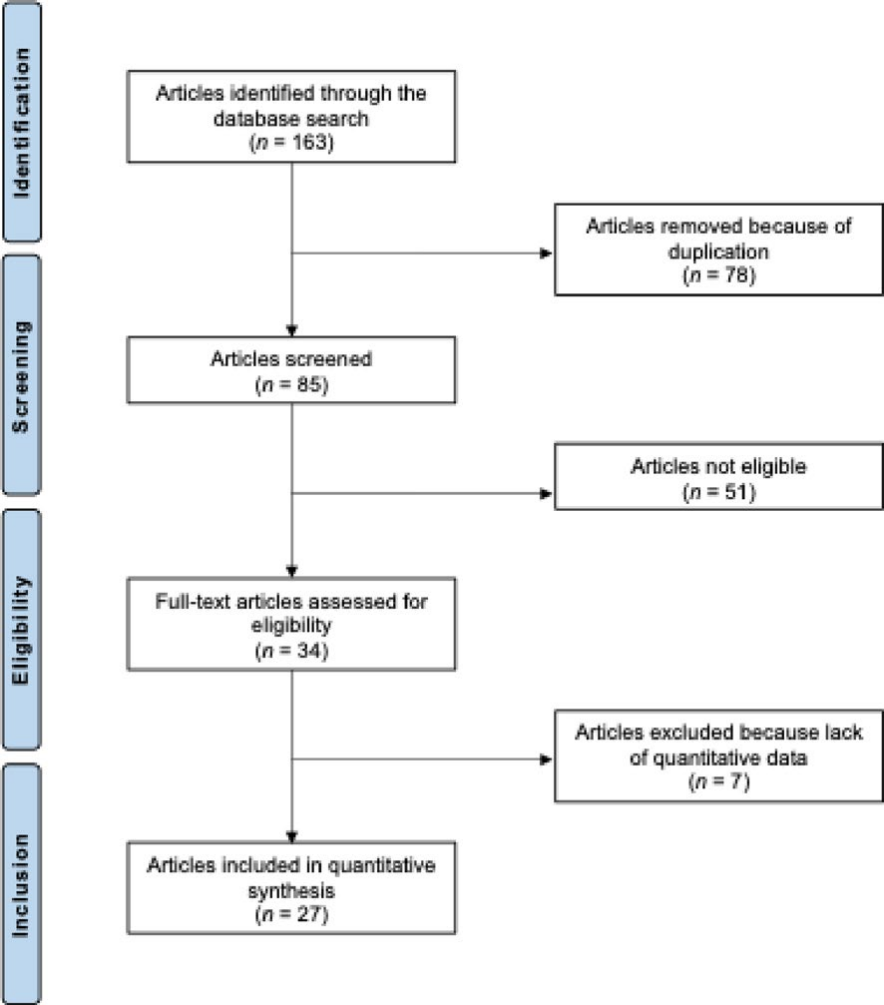
PRISMA flow chart of the literature search

The database search identified 163 records related to medial pivot TKA. After the removal of 78 duplicates, 85 records remained for screening. Titles and abstracts were reviewed, leading to the exclusion of 51 studies. The main reasons for exclusion included improper study design (N = 11), low level of evidence (N = 8), studies without a clear statement on PCL ligament management (N = 17), insufficient reporting of clinical outcomes (N = 10), and language restrictions (N = 5). Seven further studies were excluded after full-text review because no quantitative outcome data were available. A total of 27 studies met all inclusion criteria and were included in the analysis, comprising three RCTs, four prospective and 20 retrospective comparative and non-comparative series. The study selection process is illustrated in Fig. 1.

### Methodological quality assessment

**Fig. 2:**
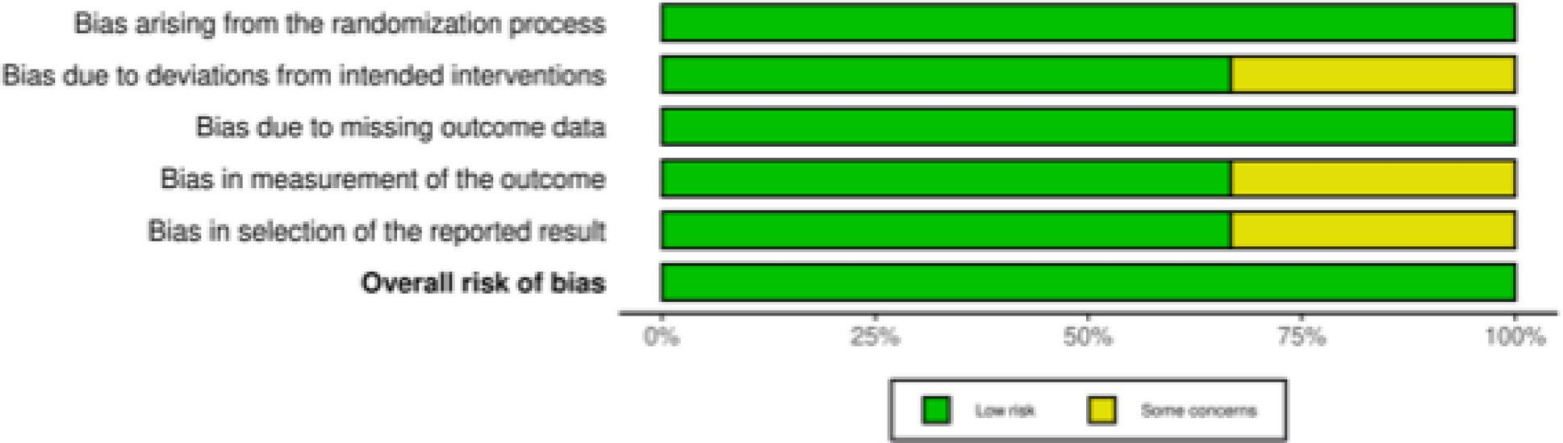
The ROB2 of RCTs

The risk of bias for RCTs included in this systematic review and meta-analysis was evaluated using the revised Risk of Bias tool (RoB 2) [34, 35]. Overall, the three trials were judged to have a low risk of bias. Randomisation and allocation were performed appropriately, and intervention protocols were followed consistently. Outcome data were complete, with no relevant attrition. Minor concerns arose in some trials, mainly for patient-reported outcomes where blinding was not always possible, and in one case because of unclear trial registration. These limitations are unlikely to have affected the overall conclusions (Fig. 2).

**Fig. 3:**
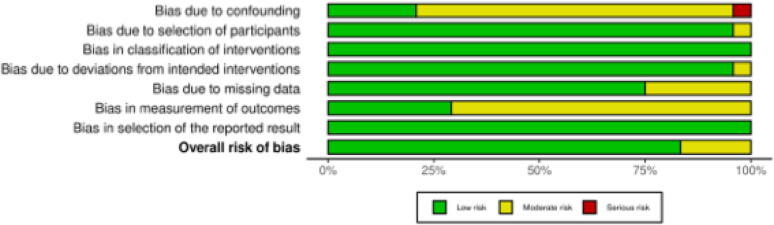
The ROBINS-I of non-RCTs

The risk of bias for the non-RCT studies was assessed using the ROBINS-I tool [37]. As expected for observational designs, most studies were considered to have a moderate risk of bias due to confounding, reflecting the absence of advanced statistical adjustment in most cases. In all other domains, the risk of bias was generally low. The majority applied clear inclusion criteria, consecutive patient recruitment, and well-described intervention protocols. Follow-up was complete or near complete in almost all studies. Measurement of outcomes, particularly patient-reported scores, was typically unblinded, in line with routine clinical practice; however, the use of standardised assessments and validated instruments minimises the impact of this limitation. Overall, the non-randomised evidence base can be considered methodologically robust, with the overall risk of bias across studies rated to be low to moderate (Fig. 3).

### Study characteristics and results of individual studies

A total of 3,380 patients were included in the present study. Of these, 2,171 underwent medial pivot TKA with resection of the PCL, while in 1,209 cases, the ligament was retained. The mean age across all patients was 70.7 ± 4.5 years. Overall, 18.6% (630 of 3380) of the patients were male. BMI was reported in most studies, with a pooled mean of 27.5 ± 2.4 kg/m^2^. The mean follow-up duration was 63.8 ± 47.3 months. A summary of study characteristics and patient demographics is presented in Table 2.

**Table 2:** Generalities and patient characteristics of the included studies

Author	Journal	LoE	Patients (n)	Male (n)	Age (y)	BMI (kg/m^2^)	Follow-up (month)	PCL	Alignment
Bae et al. [9]	*JOA*	III	67	1	67.5	26.0	47	Retained	MA
		III	70	6	65.8	26.7	47	Resected	
Batra et al. [40]	*CiOS*	IV	45	5	61.7	28.4	60	Resected	MA
Budhiparama et al. [30]	*KSSTA*	I	33	9	71.0	25.8	48	Retained	MA
		I	33	9	71.0	25.8	48	Resected	
Chinzei et al. [41]	*The Knee*	IV	85	5	70.2	26.5	93	Resected	MA
Choi et al. [42]	*JOA*	III	49	6	66.7	27.6	60	Resected	MA
Delman et al. [43]	*KSSTA*	III	25	12	70.0	29.0	19	Resected	unKA
Fan et al. [44]	*JOA*	IV	59	13	65.1		65	Resected	MA
Howel et al. [45]	*KSSTA*	III	117	52	68.0	30.0	24	Retained	unKA
Hu et al. [46]	*Heliyon*	III	84	–	68.2	27.9	103	Retained	MA
		III	168	–	67.5	28.1	105	Resected	
Ishida et al. [47]	*KSSTA*	II	71	14	71.0	27.2	57	Resected	MA
Jeremìc et al. [48]	*OTSR*	III	24	–	70.7	30.6	12	Resected	KA
		III	24	–	82.5	30.0	12	Resected	MA
Kage et al. [49]	*J. Of experimental Orthopaedic*	III	19	4	74.7	25.5	12	Resected	MA
		III	19	5	77.2	26.2	12	Resected	
Karahan et al. [50]	*Acta Orthop Belg*	III	227	40	66.6	–	79	Retained	MA
		III	77	13	66.6	–	79	Resected	
Macheras et al. [51]	*The Knee*	III	176	60	73.0	28.9	182	Retained	MA
		III	149	50	80.0	30.1	182	Resected	
Nakamura et al. [52]	*BMC*	III	45	7	74.3	25.6	24	Retained	MA
Niesen et al. [53]	*Acta Orthop*	III	35	19	67.0	31.0	12	Retained	unKA
		III	35	21	68.0	31.0	12	Retained	
Obada et al. [54]	*International Orthopaedics*	I	300	–	68.4	28.5	24	Resected	MA
Razick et al. [55]	*JEO*	IV	313	131	69.0	30.0	30	Retained	unKA
Sosio et al. [56]	*JCM*	IV	55	23	71.5	29.3	24	Resected	unKA
Ueyama et al. [57]	*Arthroplasty*	IV	96	6	70.2	27.2	142	Resected	MA
Ueyama et al. [58]	*AOTS*	III	153	18	77.0	23.0	107	Resected	MA
		III	153	23	76.0	24.0	60	Resected	
Ueyama et al. [59]	*JOA*	III	257	17	76.2	23.4	120	Resected	MA
		III	77	10	74.6	23.3	120	Retained	
Vecchini et al. [60]	*The Knee*	IV	160	42	71.0	–	84	Resected	MA
Youm et al. [61]	*Knee Surgery and Related Research*	III	80	9	66.4	–	65	Resected	MA

LoE: level of evidence; BMI: body mass index; PCL: posterior cruciate ligament; MA: Mechanical Alignment; unKA: unrestricted Kinematic Alignment

### Baseline comparability

The mean age was comparable across the cohorts (*P* = 0.149). The KSS and the BMI were comparable (*P* = 0.09 and *P* = 0.1, respectively). The proportion of men was higher in the resection cohort (*P* < 0.0001). Follow-up duration differed between groups (*P* < 0.001). These results are shown in Table 3.

**Table 3:** Baseline comparability (BMI: body mass index; KSS: Knee Society Score)

Variable	Retention (N = 1.209)	Resection (N = 2.171)	*P*-value
Age (years)	68.3 ± 7.8	68.8 ± 8.2	0.149
Sex (men, %)	18.8%	23.4%	< 0.001
BMI (kg/m^2^)	28.87 ± 3.2	27.89 ± 3.1	0.1
Follow-up (months)	50.7 ± 24.3	88.9 ± 27.5	< 0.001
KSS	83.4 ± 8.5	82.4 ± 8.9	0.090

### Results syntheses

At the last follow-up, KSS was marginally higher in the retention cohort (MD 0.98 points; *P* = 0.03), a difference that was statistically significant but not clinically meaningful. No between-group difference was detected for KSS-F (*P* = 0.4), FJS (*P* = 0.8), or OKS (*P* = 0.9). WOMAC favoured the retention cohort (MD − 8.06 points; *P* < 0.001). ROM was higher in the resection cohort (MD 5.19°; *P* < 0.001). The revision rate was low and similar between groups (OR 0.51, 95% CI 0.11–2.47; *P* = 0.3), indicating no clear difference in early survivorship. These results are shown in greater detail in Table 4.

**Table 4:** Result syntheses

Outcome	Retention (N = 1.209)	Resection (N = 2.171)	MD / OR (95% CI)	*P*-value
KSS	91.8 ± 6.7	90.8 ± 7.2	0.98 (0.09–1.87)	0.03
KSS-F	82.5 ± 7.1	81.2 ± 7.8	1.31 (− 1.52–4.14)	0.4
FJS	72.1 ± 9.8	71.8 ± 10.1	0.29 (− 2.00–2.58)	0.8
OKS	41.6 ± 5.7	41.4 ± 6.2	0.23 (− 4.52–4.98)	0.9
WOMAC	18.4 ± 6.1	26.5 ± 7.4	− 8.06 (− 10.20– − 5.92)	< 0.001
ROM (°)	122.4 ± 7.2	127.6 ± 8.1	− 5.19 (− 6.70– − 3.68)	< 0.001
Revision rate	2/1209 (0.17%)	7/2171 (0.32%)	0.51 (0.11–2.47)	0.3

KSS: Knee Society Score; KSS-F: KSS functional subscale; WOMAC: Western Ontario and McMaster Universities Osteoarthritis Index; FJS: Forgotten Joint Score; OKS: Oxford Knee Score; ROM: Range of Motion; SE: Standard Deviation; CI: Confidence Interval; MD: mean difference; OR: odds ratiome

### Meta-analyses

**Fig. 4:**
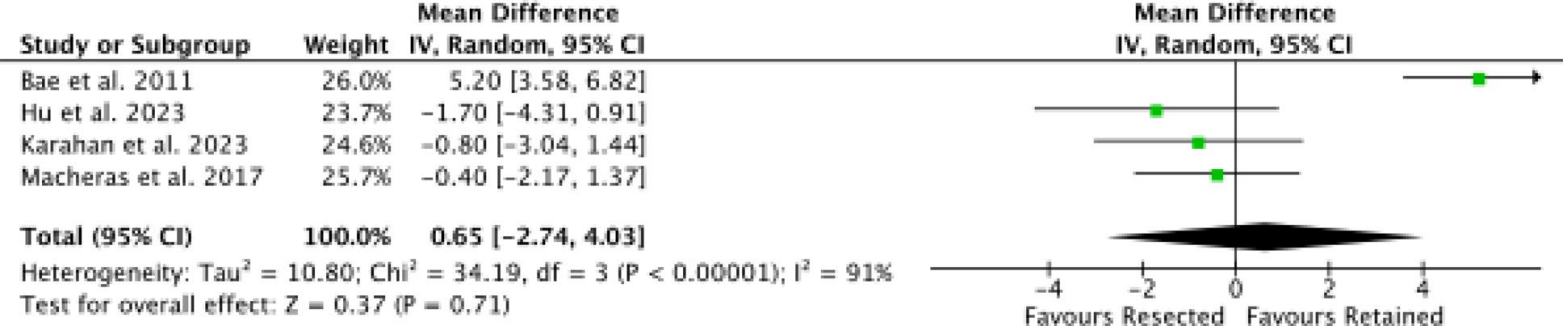
Meta-analysis of the comparison: KSS

Four studies [9, 46, 50, 51] reported data on KSS, with no difference in the effect size (*P* = 0.7, Fig. 4).

**Fig. 5:**
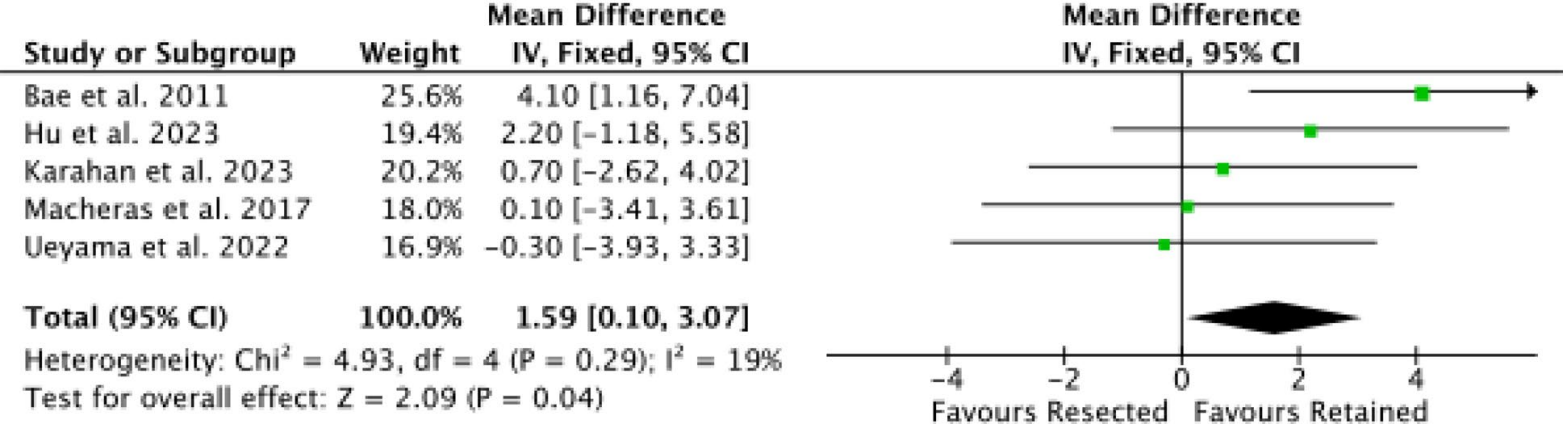
Meta-analysis of the comparison: KSS-F

Five studies [9, 46, 50, 51, 59] reported data on KSS-F, with the retention group showing a statistically significantly higher score (MD 1.59; 95% CI 0.10–3.07; *P* = 0.04; Fig. 5).

**Fig. 6:**
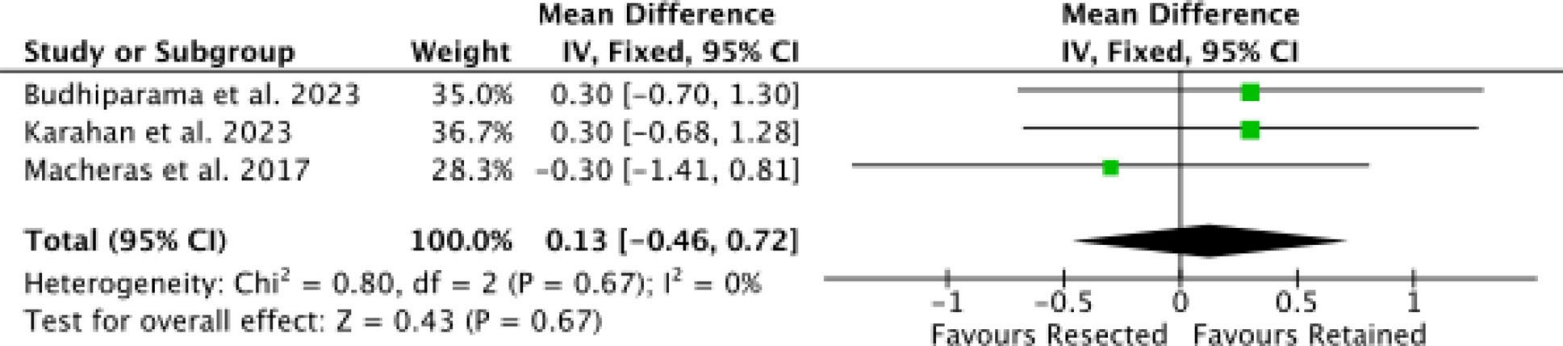
Meta-analysis of the comparison: OKS

Three studies [30, 50, 51] reported data on OKS, with no difference in the effect size (*P* = 0.7, Fig. 6).

**Fig. 7:**
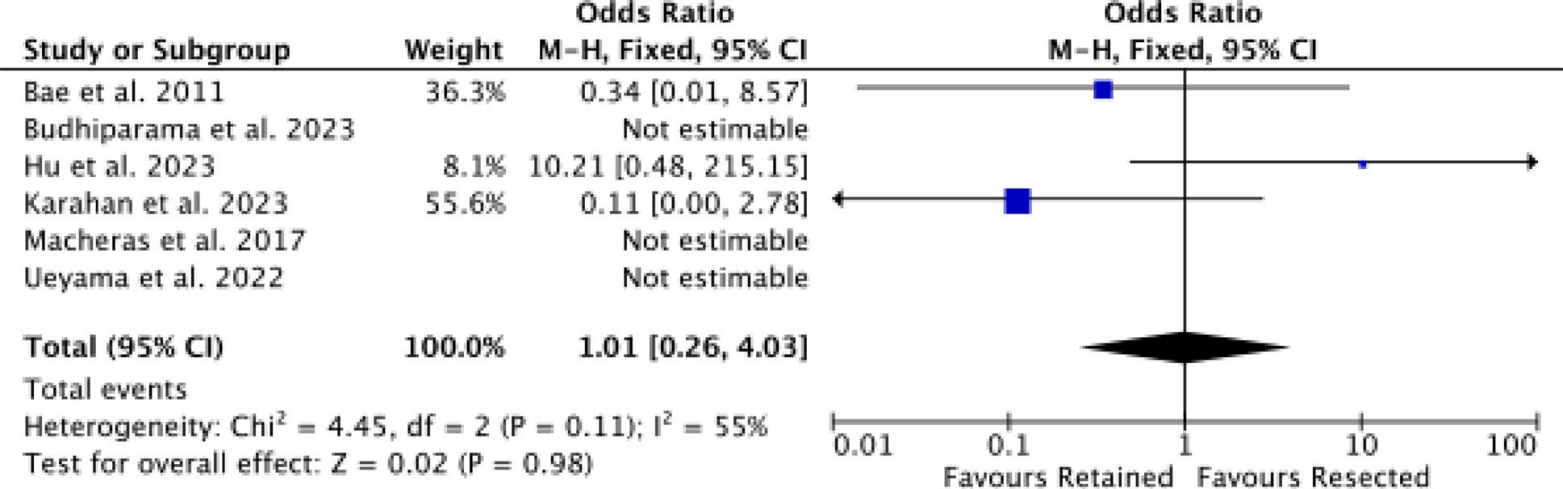
Meta-analysis of the comparison: revision score

Six studies [9, 30, 46, 50, 51, 59] reported data on revision rate, with no difference in the effect size (*P* = 0.9, Fig. 7).

**Fig. 8:**
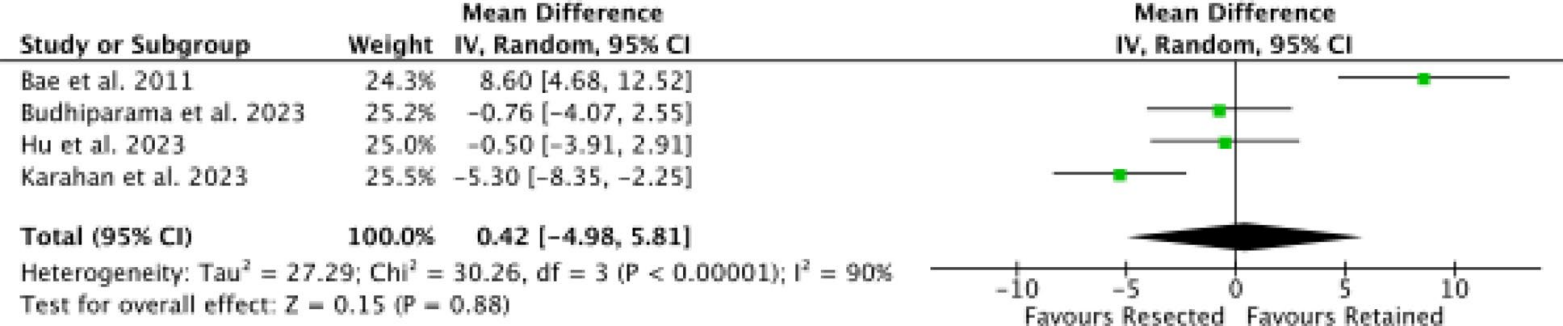
Meta-analysis of the comparison: ROM

Four studies [9, 30, 46, 50] reported data on ROM, with no difference in the effect size (*P* = 0.9, Fig. 8).

## Discussion

The present meta-analysis revealed no clinically meaningful differences between posterior cruciate ligament (PCL) retention and resection in medial pivot total knee arthroplasty (MP-TKA). Although some functional scores, such as KSS and WOMAC, showed statistically higher values in the PCL-retained cohort, these differences remained below the corresponding minimal clinically important difference (MCID) thresholds. Hence, while the data may suggest a slight numerical advantage in favour of PCL retention, this finding is unlikely to reflect a clinically perceptible improvement. The same applies to the small range-of-motion advantage observed in the resected group, which, although statistically significant, did not reach clinical relevance. Surgical decisions regarding PCL management should therefore be guided by intraoperative balancing, surgeon experience, and individual patient factors rather than the expectation of superior outcomes. Nevertheless, the interpretation of the results should be cautious. The baseline comparability analysis revealed differences in follow-up duration and sex distribution between the cohorts, which could serve as potential confounders of the observed outcomes. These methodological imbalances limit the strength of inference and highlight the need for future studies with more homogeneous populations, standardised follow-up, and well-matched baseline characteristics to provide more reliable comparative evidence.

The biomechanical rationale behind this small divergence deserves attention. Retention of the posterior cruciate ligament has been demonstrated in cadaveric and kinematic studies to allow for greater internal tibial rotation during deep flexion, nearly doubling the passive motion compared with resection [18, 21, 62, 63]. This increase in rotational capacity may result in a smoother flexion arc, which could explain the slightly higher functional scores observed, even though these do not meet clinical thresholds of significance [22, 64]. In addition, computational analyses have reported that preserving the ligament maintains higher tibiofemoral contact forces while reducing patellofemoral loading compared with excision, a factor that may reduce anterior knee discomfort and favour quadriceps function [65, 66]. The influence of ligament preservation on stability has also been investigated. Robotic studies that measured gap tensioning revealed that posterior cruciate ligament resection increased mediolateral laxity during flexion by small but measurable amounts, with values ranging from one to two millimetres [67, 68]. This might suggest a predisposition to subtle instability, particularly in valgus knees; however, the magnitude of change was not sufficient to impact functional outcomes in a clinically relevant way [22, 69]. Computational models confirmed that resection altered load distribution between compartments, particularly reducing medial compartment forces, but this redistribution did not manifest as a disadvantage in clinical follow-up [22, 65, 66]. From a biological perspective, preservation of the ligament may retain native proprioceptive structures and mechanoreceptors that contribute to joint position sense and neuromuscular control [70–75]. Although these potential benefits are difficult to quantify in clinical practice, they provide a theoretical basis for the slightly improved functional scores recorded in the retention group. Nevertheless, evidence from large clinical series and previous meta-analyses consistently indicates that patient-reported outcomes, gait parameters, and overall satisfaction remain comparable regardless of cruciate management [22, 76, 77]. Studies examining patient awareness of prosthesis type have further shown that individuals are generally unable to perceive whether the ligament has been sacrificed or preserved, reinforcing the equivalence of both strategies in practical terms [20, 78, 79]. In conclusion, while posterior cruciate ligament retention demonstrated a statistically significant advantage in the functional subscale of the KSS, this benefit did not exceed the minimal clinically important difference and therefore cannot be regarded as clinically relevant. The biomechanical rationale of improved rotational kinematics and more physiological load transfer supports the concept of retention, yet the biological contribution of proprioceptive preservation remains speculative [80, 81]. Surgical decision-making should therefore not be guided by the expectation of substantial functional superiority of one technique over the other but rather by individual anatomical characteristics, intraoperative stability, and surgeon expertise.

This review has several limitations that should temper interpretation. Most included studies are non-randomised and retrospective. Consequently, confounding by indication remains a likely concern: surgeons may decide to retain or resect the PCL based on intraoperative stability, bone quality, or personal preference, which can bias outcomes independently of the implant philosophy. True head-to-head comparisons are relatively few, and several have imbalances in group size, which reduces power and increases susceptibility to type I/II errors. There is meaningful heterogeneity across medial-pivot generations. The evidence base mixes first-generation MP designs with second-generation designs that refined medial conformity, lateral condylar geometry, trochlear groove anatomy, polyethylene characteristics, and insert constraint options. Earlier MP constructs generally emphasised a highly conforming medial compartment with comparatively simpler lateral geometry; when the PCL is resected in such designs, femoral rollback and mid-flexion stability may depend more on articular conformity alone [82]. Newer second-generation MP systems often incorporate updated lateral geometry and patellofemoral features intended to reduce paradoxical anterior translation, enhance mid-flexion stability, and improve tracking; some also offer inserts specifically optimised for PCL-retained or PCL-resected strategies [58]. Pooling these different generations likely dilutes design-specific effects and makes any observed differences between PCL retention and resection partly design-dependent rather than purely “strategy-dependent.” Surgical technique variability is considerable and rarely standardised: targets for alignment, balancing philosophy (measured resection vs gap-balancing), tibial slope, patellar resurfacing policies, soft-tissue release thresholds, and rehabilitation protocols differ across studies and are often incompletely reported. These factors interact with both MP design and PCL management, complicating causal inference. In addition, alignment philosophy differed across studies: most used conventional mechanical alignment, whereas only a minority applied unrestricted kinematic alignment. As alignment influences both MP kinematics and the functional role of the PCL, this heterogeneity may have contributed to variation in reported outcomes. None of the included papers reported robotic- or navigation-assisted procedures. Follow-up duration is heterogeneous, with some series providing long-term data, while others are limited to short- or mid-term assessments. The revision outcomes summarised in our table include only mechanical endpoints, aseptic loosening and revision for instability, to focus on implant mechanics. Revisions for septic complications or trauma were intentionally excluded; while methodologically coherent with our objective, this choice narrows external validity and may underestimate overall revision burden. Collectively, these limitations argue for cautious interpretation of between-group differences and support the need for well-powered, design-specific, prospectively registered comparative studies with standardised techniques and endpoints. Future studies should be randomised, comparing PCL retention versus resection within the same MP design and generation, with standardised alignment and tibial slope. Measure stability with sensors or instrumented tests, including kinematic/RSA sub-studies, and report core outcomes at fixed times.

## Conclusion

PCL retention and resection in medial pivot TKA yield statistically different but clinically equivalent results. The small numerical advantages observed for the retained group in certain functional outcomes do not appear to represent a meaningful clinical improvement. Both strategies can therefore be considered viable, and adequate alignment, balancing, and soft-tissue management remains pivotal. Further high-quality comparative studies involving well-matched populations are warranted to clarify whether subtle functional trends associated with PCL retention have consistent long-term clinical significance.

## Data Availability

The datasets generated during and/or analysed during the current study are available throughout the manuscript.

## Declarations

### Use of AI

Language editing was performed using Grammarly (Superhuman Platform Inc., San Francisco, CA, USA).

### Registration and protocol

The present review was not prospectively registered, as it was conducted retrospectively based on a predefined search strategy and available dataset.
